# Measurement reproducibility of slice-interleaved T_1_ and T_2_ mapping sequences over 20 months: A single center study

**DOI:** 10.1371/journal.pone.0220190

**Published:** 2019-07-25

**Authors:** Jihye Jang, Long H. Ngo, Gabriella Captur, James C. Moon, Reza Nezafat

**Affiliations:** 1 Department of Medicine (Cardiovascular Division), Beth Israel Deaconess Medical Center and Harvard Medical School, Boston, MA, United States of America; 2 Department of Computer Science, Technical University of Munich, Munich, Germany; 3 Department of Biostatistics, Harvard T.H. Chan School of Public Health, Boston, MA, United States of America; 4 Barts Heart Center, The Cardiovascular Magnetic Resonance Imaging Unit, St Bartholomew’s Hospital, West Smithfield, London, United Kingdom; 5 NIHR University College London Hospitals Biomedical Research Center, London, United Kingdom; 6 UCL Institute of Cardiovascular Science, University College London, London, United Kingdom; New York University School of Medicine, UNITED STATES

## Abstract

**Background:**

Quantifying reproducibility of native T_1_ and T_2_ mapping over a long period (> 1 year) is necessary to assess whether changes in T_1_ and T_2_ over repeated sessions in a longitudinal study are associated with variability due to underlying tissue composition or technical confounders.

**Objectives:**

To carry out a single-center phantom study to 1) investigate measurement reproducibility of slice-interleaved T_1_ (STONE) and T_2_ mapping over 20 months, 2) quantify sources of variability, and 3) compare reproducibility and measurements against reference spin-echo measurements.

**Methods:**

MR imaging was performed on a 1.5 Tesla Philips Achieva scanner every 2–3 weeks over 20 months using the T1MES phantom. In each session, slice-interleaved T_1_ and T_2_ mapping was repeated 3 times for 5 slices, and maps were reconstructed using both 2-parameter and 3-parameter fit models. Reproducibility between sessions, and repeatability between repetitions and slices were evaluated using coefficients of variation (CV). Different sources of variability were quantified using variance decomposition analysis. The slice-interleaved measurement was compared to the spin-echo reference and MOLLI.

**Results:**

Slice-interleaved T_1_ had excellent reproducibility and repeatability with a CV < 2%. The main sources of T_1_ variability were temperature in 2-parameter maps, and slice in 3-parameter maps. Superior between-session reproducibility to the spin-echo T_1_ was shown in 2-parameter maps, and similar reproducibility in 3-parameter maps. Superior reproducibility to MOLLI T_1_ was also shown. Similar measurements to the spin-echo T_1_ were observed with linear regression slopes of 0.94–0.99, but slight underestimation. Slice-interleaved T_2_ showed good reproducibility and repeatability with a CV < 7%. The main source of T_2_ variability was slice location/orientation. Between-session reproducibility was lower than the spin-echo T_2_ reference and showed good measurement agreement with linear regression slopes of 0.78–1.06.

**Conclusions:**

Slice-interleaved T_1_ and T_2_ mapping sequences yield excellent long-term reproducibility over 20 months.

## Introduction

Cardiovascular magnetic resonance (CMR) native T_1_ and T_2_ mapping have emerged as promising techniques for myocardial tissue characterization [1]. Studies have reported increased native T_1_ times in the presence of myocardial fibrosis, inflammation, amyloids, and decreased T_1_ in the presence of Anderson-Fabry disease, and iron overload [2]. Increased T_2_ times have also been reported in the presence of edema or inflammation [3–5]. Assessing T_1_ and T_2_ measurement reproducibility is a necessary step toward their clinical utility as quantitative imaging biomarkers [6].

Various cardiac mapping techniques have been proposed for T_1_ [7–11] and T_2_ mapping [12–16]. The most widely used T_1_ mapping sequence is the Modified Look-Locker inversion recovery (MOLLI) [7], which is based on sampling the inversion recovery of the longitudinal relaxation signal. Other types of T_1_ mapping sequences, such as the Saturation recovery single-shot acquisition (SASHA), are based on sampling the saturation recovery curve [9]. A hybrid sequence combining inversion and saturation recovery curves, such as the Saturation pulse prepared heart rate independent inversion recovery (SAPPHIRE), has also been proposed [10]. The most widely used T_2_ mapping sequences are based on T_2_-preparation (T_2_prep) [17–20] with balanced steady-state free precession (bSSFP) imaging [5, 12] or spoiled gradient echo (GRE) [14] acquired with at least 3 different echo times. Other types of T_2_ mapping sequences are based on turbo spin echo (TSE) [15] or gradient spin echo (GraSE) [16].

In longitudinal studies, understanding technical variability is critical to determining if observed changes over time are biological and therefore clinically significant or only related to measurement variation [21]. Furthermore, higher reproducibility means fewer patients are necessary to achieve statistical significance in clinical trials, ultimately reducing study costs [22]. Several prior studies have investigated the reproducibility of various T_1_ and T_2_ mapping sequences, however they are test/retest studies carried out within several weeks [23–26]. Reproducibility studies using MOLLI and shortened MOLLI (ShMOLLI) have demonstrated that both sequences are highly reproducible [24, 26–29]. SASHA and SAPPHIRE were reported to have similar reproducibility as inversion recovery-based sequences [23]. The reproducibility of T_2_ mapping of multi-echo-spin-echo T_2_, T_2_prep-bSSFP, and GraSE T_2_ mapping sequences were also reported to be excellent [25].

The free-breathing slice-interleaved T_1_ [30, 31] and T_2_ [32] mapping techniques have been proposed and used in various clinical scenarios [33–37]. Slice-interleaved T_1_ (STONE) acquires data for different slices within one inversion recovery curve to allow more accurate measurement with a bSSFP (STONE-bSSFP) [30] or spoiled gradient echo (STONE-GRE) [31]. Slice-interleaved T_2_ uses slice-selective T_2_prep with an interleaved slice acquisition scheme which permits increased time efficiency [32]. Slice-interleaved T_1_ and T_2_ mapping sequences provide highly reproducible measurements in test/retest studies of healthy subjects [22], however the long-term reproducibility (> 1 year) has not yet been studied. Long-term reproducibility of T_1_ and T_2_ measurements using slice-interleaved T_1_ and T_2_ mapping needs to be investigated prior to utilization of these sequences in longitudinal studies monitoring disease progression or treatment efficacy.

Various confounders can impact the accuracy and reproducibility of myocardial tissue characterization. Therefore, performance assessment of the myocardial tissue characterization techniques requires rigorous in-vivo or phantom validation. While in-vivo studies are the ideal experimental setting, a phantom study is necessary in cases where in-vivo experiments are not feasible or scenarios in which the reference standard can only be measured in a phantom setting. Phantom studies are also necessary for assessing long-term measurement variability when scanning volunteers for extended periods over multiple sessions is not feasible. T_1_ or T_2_ accuracy and temperature sensitivity, for example, can only be measured in the phantom setting. Although a phantom experiment may not address all relevant confounding factors of an in-vivo setting, it provides valuable information that may not be easily attainable from an in-vivo experiment.

The aim of this study was to carry out a single-center phantom study to 1) investigate the measurement reproducibility of slice-interleaved T_1_ and T_2_ mapping over 20 months, 2) quantify sources of variability, and 3) compare the performance of each in terms of reproducibility and measurement against reference spin-echo measurements.

## Materials and methods

**Fig 1:**
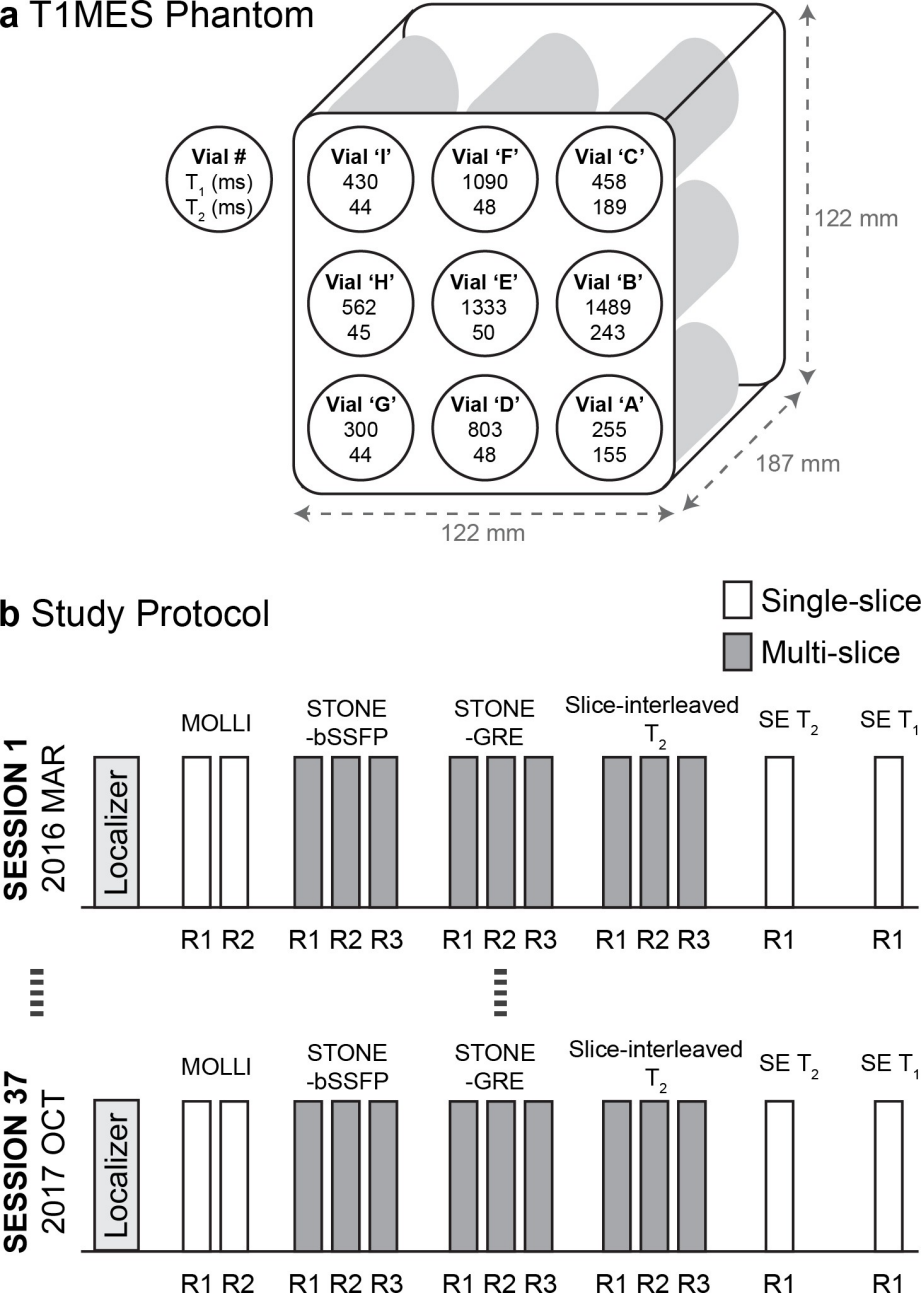
T1MES phantom used in this study, and the reproducibility study protocol. a) The T1MES phantom used in this study consists of 9 vials of NiCl_2_ doped agarose covering T_1_ and T_2_ ranges in the blood and myocardium before and after Gadolinium-based contrast agents. b) An imaging session was repeated every 2–3 weeks over 20 months (between-session reproducibility). Within each session, slice-interleaved T_1_ and T_2_ mapping sequences were repeated 3 times (between-repetition repeatability) for five slices (between-slice repeatability). SE T_1_ and T_2_ measurements and MOLLI were performed for comparison. STONE-bSSFP, slice-interleaved T_1_ with balanced steady‐state free precession; STONE-GRE, slice-interleaved T_1_ with spoiled gradient echo; SE, spin-echo.

Experiments were performed using T_1_ Mapping and ECV Standardization Program (T1MES) phantom [38]. This Food and Drug Administration (FDA)-cleared/Conformité Européene (CE)-marked MR phantom enables stable quality measures to study measurement variability over time. T1MES contains 9 vials (NiCl_2_ doped agarose) covering the physiological ranges of T_1_ and T_2_ in the blood and myocardium pre- and post-Gadolinium-based contrast agents (GBCA; for a 1.5 T phantom: T_1_:255ms to 1489ms, T_2_:44ms to 243ms, referenced from the T1MES manual measured by slow inversion-recovery/spin-echo methods at 1.5T) (Fig 1A). The T1MES phantom volume is 2L with an inner dimension size of 197 × 122 × 122 mm, and the vials have a minimum diameter of 20 mm [38]. For T_1_ mapping, all 9 vials were studied given that the phantom is designed to include all relevant T_1_ ranges of myocardium and blood pre- and post-GBCA. For T_2_ mapping we only studied vial ‘F’ (Fig 1A) which modulates “Medium” native myocardial T_1_ and T_2_ times at 1.5 T. Our T_2_ mapping sequence is not designed to handle high T_2_ values over 100 ms found in the blood, and all remaining vials had no variability (44–50 ms).

Reproducibility is defined as the measurement precision between replicate measurements under varying conditions, and repeatability is defined as the measurement precision between replicate measurements under constant conditions [21]. In this study, we use ‘reproducibility’ when referring to measurement precision over multiple sessions, and ‘repeatability’ when referring to scanning in the same session. We defined a ‘session’ as a ‘single CMR imaging with identical image localization’.

The study design schematic is shown in Fig 1B. Reproducibility over several weeks was reported for between-session reproducibility. Images were acquired using STONE-bSSFP T_1_ [30], STONE-GRE T_1_ mapping [31], and slice-interleaved T_2_ mapping [32] sequences. Within each session, imaging was repeated 3 times to allow repeatability assessment within each session and between repetitions. For multi-slice sequences, between-slice repeatability was also studied. Additionally, we acquired spin-echo (SE) T_1_ and T_2_ measurements and MOLLI in each imaging session for comparison; MOLLI was repeated 2 times.

### CMR imaging

CMR imaging was performed using a 1.5 T scanner (Philips Achieva, Best, The Netherlands) with a 32-element cardiac phased-array receiver coil. The phantom was stored and scanned at room temperature in the scanner room. We assumed temperature and subsequently diffusion was uniform along vials in our study. Scanning was strictly performed according to the T1MES phantom user manual [38]. All acquisitions were performed with a simulated electrocardiogram (ECG) at a RR (interval time between two R-waves) period of 900 ms (heart rate 67 bpm). The positioning process was consistent for all sessions throughout the study. The book used to lift the phantom, large towel, coil, software version of the scanner, and air-flow setting of the scanner room remained constant throughout the study.

#### T_1_ mapping

The STONE-bSSFP sequence was acquired with the following parameters: 5 slices, in-plane resolution = 2.1 × 2.1 mm^2^, slice thickness = 8 mm, slice gap = 4 mm, field-of-view = 280 × 280 mm^2^, TR/TE/flip angle = 2.8 ms / 1.39 ms / 70°, a sensitivity encoding (SENSE) rate = 2, linear ordering, 10 linear ramp-up pulses and bandwidth = 1894 Hz, acquisition duration = 1 min 38 sec. Eleven inversion images were acquired with inversion times of ∞, 130, 1030, 1930, 2830, 3730, 350, 1250, 2150, 3050, and 3950 ms. The STONE-GRE sequence was acquired with the following parameters: 5 slices, in-plane resolution = 2 × 2 mm^2^, slice thickness = 8 mm, slice gap = 4 mm, field-of-view = 280 × 280 mm^2^, TR/TE/flip angle = 4.7 ms / 2.3 ms / 10°, a SENSE rate = 2.5, half-scan factor = 0.75, linear ordering, 10 linear ramp-up pulses and bandwidth = 383 Hz, acquisition duration = 1 min 38 sec. Eleven inversion images were acquired with inversion times of ∞, 109, 1009, 1909, 2809, 3709, 350, 1250, 2150, 3050, and 3950 ms. For both STONE-bSSFP and STONE-GRE sequences, the inversion preparation pulse was an adiabatic hyperbolic secant pulse with an 11 ms pulse duration. The radiofrequency (RF) excitation pulse was a slice-selective Sinc-Gauss pulse with a duration of 0.43 ms. Images were acquired without prospective slice tracking, and the order of slices was 1-4-2-5-3. The MOLLI 5b(3s)3b [39] sequence was acquired with the following parameters: single slices, in-plane resolution = 2 × 2 mm^2^, slice thickness = 8 mm, field-of-view = 280 × 280 mm^2^, TR/TE/flip angle = 2.6 ms / 1.30 ms / 35°, a SENSE rate = 2.5, linear ordering, 10 linear ramp-up pulses and bandwidth = 1786 Hz, acquisition duration = 8 sec. Eight inversion images were acquired with inversion times of 79, 979, 1879, 2779, 3679, 350, 1250, and 2150 ms. SE T_1_ times were obtained using inversion-recovery SE acquisitions with 16 inversion times of 50, 100, 200, 300, 400, 500, 600, 700, 800, 900, 1000, 1250, 1500, 1750, 2000, and 3000 ms with the following imaging parameters: single slice, in-plane resolution = 1.2 × 1.2 mm^2^, slice thickness = 8 mm, field-of-view = 140 × 140 mm^2^, TR/TE/flip angle = 10 s / 11 ms / 90° and bandwidth = 510 Hz, acquisition duration = 5 hour 18 min.

#### T_2_ mapping

The slice-interleaved T_2_ mapping sequence was acquired with the following parameters: 5 slices, in-plane resolution = 2 × 2 mm^2^, slice thickness = 8 mm, slice gap = 4 mm, slice ordering = 1-3-5-2-4, field-of-view = 280 × 280 mm^2^, TR/TE/flip angle = 2.8 ms / 1.42 ms / 55°, a SENSE rate = 2.5, linear ordering, 10 linear ramp-up pulses and bandwidth = 1786 Hz, acquisition duration = 1 min 26 sec. Ten T_2_prep images were acquired with T_2_prep echo times of 0, 25, 35, 45, 55, 65, 75, 85, 95 ms, and ∞ was simulated with a saturation pulse. For T_2_ mapping with 4 echo times, T_2_prep images of 0, 25, 55, and ∞ were used for map reconstruction, and the results are reported as T_2_ 4echo. A T_2-_prep pulse consists of a tip-down slice-selective 90° pulse, followed by four non-selective 180° refocusing pulses that end with a closing tip-up slice-selective 90° pulse [32, 40]. SE T_2_ times were obtained using a Carr-Purcell-Meiboom-Gill (CPMG) SE sequence with 32 TEs of 10, 20, 30, …, 320 ms. The imaging parameters were as follows: single slice, in-plane resolution = 1.16 × 1.16 mm^2^, slice thickness = 8 mm, field-of-view = 140 × 140 mm^2^, TR/TE/flip angle = 10 s / 10 ms / 90°, number of signals averaged = 4, bandwidth = 1029 Hz, acquisition duration = 1 hour 21 min.

### Map reconstruction

Slice-interleaved T_1_ and T_2_ maps were reconstructed using both 2-parameter (2P) and 3-parameter (3P) fit models and all results were reported for both 2P and 3P maps. T_1_ and T_2_ maps were reconstructed offline using MATLAB (MathWorks Inc., Natick, Massachusetts, USA). STONE-bSSFP and STONE-GRE maps were estimated by voxel-wise curve-fitting of the signal with a 2-parameter ($S_{T_{1},2P}$) and 3-parameter ($S_{T_{1},3P}$) model of the inversion-recovery signal [30]. MOLLI and SE T_1_ values were obtained using $S_{T_{1},3P}$. For MOLLI, apparent T_1_ values were corrected using Look-Locker correction based on the fitted parameters [7].

For slice-interleaved T_2_ mapping, a 2-parameter ($S_{T_{2},2P}$) and 3-parameter ($S_{T_{2},3P}$) curve fitting model of the T_2_ signal capturing the effect of imaging pulses on the magnetization was used [41]. SE T_2_ values were estimated using $S_{T_{2},2P}$. All parameters were estimated using a Levenberg-Marquardt optimizer [42].

### Data analysis

A region-of-interest (ROI) was manually contoured once for each vial, and identical ROIs were programmatically applied to all slice-interleaved T_1_, T_2_ and MOLLI maps throughout all experiments. A graphical illustration of the ROI is shown in S1 Fig. The mean area of the elliptical ROIs of each vial was 73 mm^2^. A separate ROI was manually contoured once and identical ROIs were used for all SE T_1_ and T_2_ maps throughout all sessions. The mean area of the elliptical SE ROIs of each vial was 90 mm^2^. A linear translation of ROIs less than 1cm in the imaging plane directions was applied in case of offsets from the isocenter. The measurement was defined for each vial as the mean T_1_ or T_2_ in each ROI and was acquired separately for all slices, repetitions, sessions, vials, and sequences. Data analysis was performed using MATLAB (MathWorks Inc., Natick, Massachusetts, USA).

### Statistical analysis

To investigate T_1_ and T_2_ measurement drift over 20 months, a linear regression was performed for each vial over sessions, and the regression slope and 95% confidence interval (CI) of the slopes were reported. We carried out three analyses to assess the reproducibility and repeatability of the observed slice-interleaved T_1_ and T_2_ measurements via coefficients of variation, variance component decompositions, linear regressions, and Bland-Altman plots.

#### Estimation of coefficient of variation

The coefficient of variation (CV), defined as the ratio of the standard deviation to the mean multiplied by 100, was performed to assess reproducibility between sessions, repeatability between repetitions and within a session, and repeatability between slices and within single repetitions. CV was reported as the mean ± standard deviation and visualized by bar plots. To further study variability in T_1_ mapping due to different T_1_ times, a CV scatter plot for each vial, sorted from shortest T_1_ to longest T_1_ time, and a Spearman correlation between the CV and T_1_ time (vials) was reported. For T_2_ mapping, between-session reproducibility CV was estimated for a single vial and therefore no standard deviation among sessions was reported. CV was considered excellent at 0–5%, and good at 5–10%.

#### Variance decomposition analysis

We considered the observed T_1_ and T_2_ measurements as random variables whose variability originates from experimental factors and measurement errors. We considered temperature, session, repetition, and slice as the experimental factors and studied how much T_1_ and T_2_ variability is due to each of these factors. Variance component decomposition analysis [43] yielded an estimation of variance components for each factor. The mean square variance and the variance component to total variance ratio was multiplied by 100, yielding the variability percentage of the respective experimental factor. The analysis was performed for each vial, and we reported the averaged variance and variance ratio of all vials respectively.

#### Performance analysis against the spin echo

For T_1_ mapping, a t-test was performed to assess between-session reproducibility differences between reference SE T_1_ measurements and MOLLI vs. slice-interleaved T_1_ sequences. Measurement comparison analysis of each sequence to the SE was also performed by using the Pearson correlation between the SE and each sequence. Linear regression was performed and slopes between the sequences and the 95% CI of the slopes were reported. Finally, Bland-Altman analysis was performed to study measurement bias between the two sequences, and the percentage of data points outside of the 95% limits of agreement (mean ± 2 standard deviations) was reported.

For T_2_ mapping, the relative CV percentage difference between slice-interleaved T_2_ and SE T_2_ was reported to assess differences in between-session reproducibility. A measurement comparison analysis to the SE was performed using the Pearson correlation, linear regression, and Bland-Altman analysis. Since only one vial was used for T_2_ mapping analyses, slice-interleaved T_2_ was averaged over all slices/ repetitions for each of the 37 sessions and compared to the SE T_2_ measurement of 37 sessions.

For all analyses, type-I error was set to 0.05. All statistical analyses were performed with SAS software (SAS Institute Inc., Cary, North Carolina, USA).

## Results

Thirty-seven imaging sessions were performed from March 7, 2016 to October 31, 2017 The interval between successive sessions was 17±4 days. One session was excluded from the analysis due to incomplete acquisition of the SE T_1_ sequence. The isocenter cross marker of the phantom bottle enabled consistent positioning of the phantom throughout the study. Linear translations of ROIs were applied in 6 sessions with the offsets from the isocenter of 2.19±1.20 mm. Examples of T_1_ and T_2_ weighted images of each sequence are shown in S2 Fig. The temperature of the scanner room over the 20 months duration of experiments was 20.22±1.12°C (range 18–22°C). No measurement drift was observed in vials with low T_1_ (<1000 ms) over the 20 month study duration; increased T_1_ measurements were observed in vials with high T_1_ (>1000 ms) (S3 Fig; S1 Table). No drift in the T_2_ measurements was observed over the 20 month study duration (S4 Fig; S2 Table).

### T_1_ Mapping

#### Estimation of coefficient of variation

**Fig 2:**
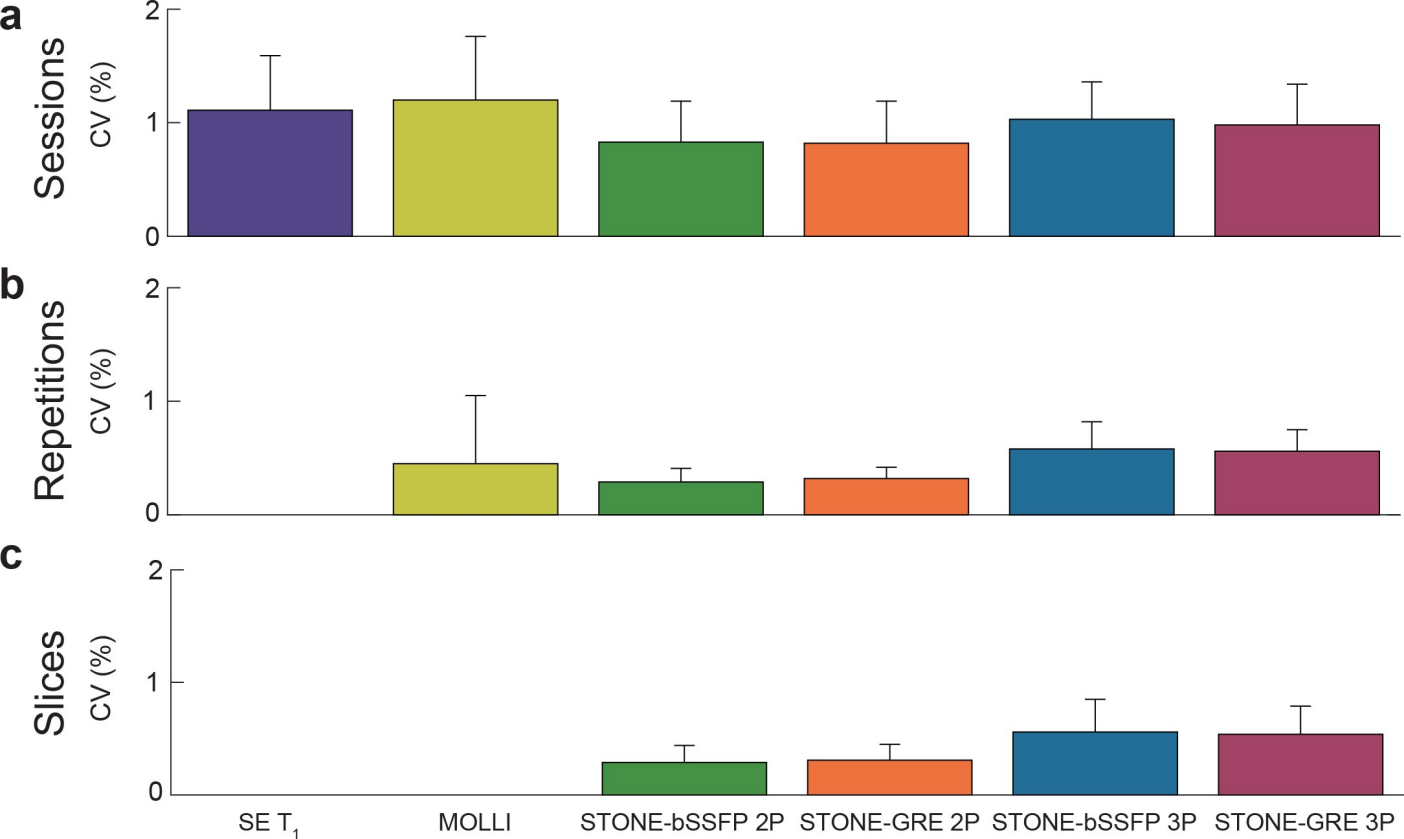
Reproducibility between sessions, and repeatability between repetitions and slices of slice-interleaved T_1_ mapping sequences were assessed using coefficients of variation (CV). Slice-interleaved T_1_ mapping sequences showed excellent between-session reproducibility (CV: SE T_1_ = 1.1±0.5%, MOLLI = 1.2±0.6%, STONE-bSSFP 2P = 0.8±0.4%, STONE-GRE 2P = 0.8±0.4%, STONE-bSSFP 3P = 1.0±0.3%, STONE-GRE 3P = 1.0±0.4%), between-repetition repeatability (CV: MOLLI = 0.5±0.6%, STONE-bSSFP 2P = 0.3±0.1%, STONE-GRE 2P = 0.3±0.1%, STONE-bSSFP 3P = 0.6±0.2%, STONE-GRE 3P = 0.6±0.2%), and between-slice repeatability (CV: STONE-bSSFP 2P = 0.3±0.2%, STONE-GRE 2P = 0.3±0.1%, STONE-bSSFP 3P = 0.6±0.3%, STONE-GRE 3P = 0.5±0.3%).

**Fig 3:**
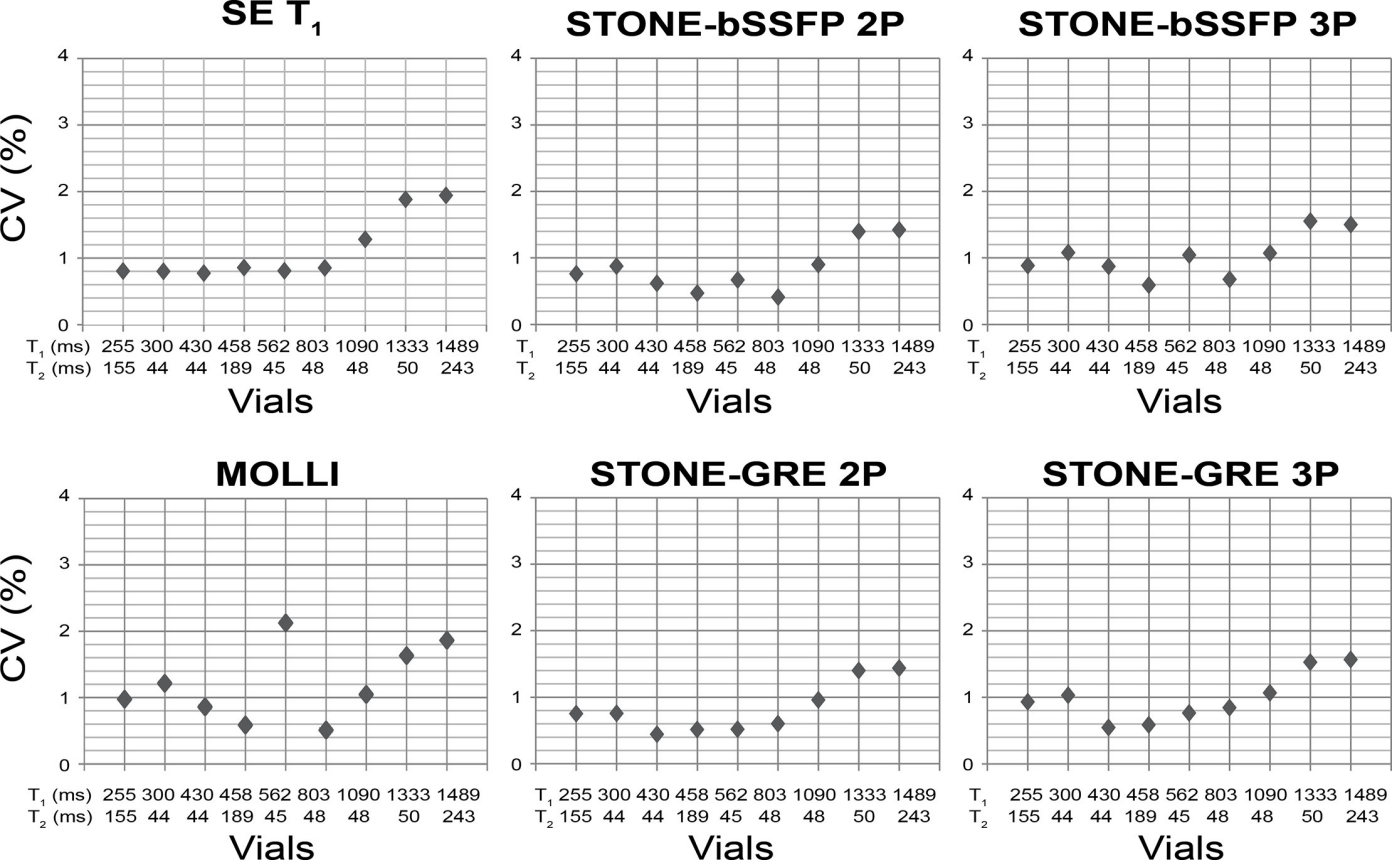
Coefficients of variations (CV) shown as scatter plots for each vial. Vials are sorted from shortest T_1_ to longest T_1_ time (reference T_1_ from the T1MES manual measured by slow inversion-recovery/spin-echo methods at 1.5T: 255, 300, 430, 458, 562, 803, 1090, 1333, and 1489 ms). Vials with higher T_1_ time show higher variability.

Excellent reproducibility between sessions, and excellent repeatability between repetitions and slices of slice-interleaved T_1_ mapping sequences were observed with a CV less than 2% (Fig 2). There was a positive association between the T_1_ value and the CV, with longer T_1_ times corresponding to higher variability (Fig 3). The Spearman correlation between the T_1_ of each vial and the variability of each sequence was as follows: SE T_1_ = 0.88, MOLLI = 0.37, STONE-bSSFP 2P = 0.48, STONE-bSSFP 3P = 0.48, STONE-GRE 2P = 0.60, and STONE-GRE 3P = 0.60.

#### Variance decomposition analysis

The sources of variability for slice-interleaved T_1_ mapping sequences are summarized in Table 1. The main source of variability was temperature when reconstructed with a 2-parameter fit model, and slice location/ orientation when reconstructed with a 3-parameter fit model. Repeated measurements within the same session at the same slice location did not contribute to variability (variance decompositions less than 1%).

**Table 1:** Sources of variability in T_1_ mapping defined by variance decomposition analysis.

T_1_ Mapping, variance [ms^2^] (variance ratio [%])						
	SE T_1_	MOLLI	STONE-bSSFP 2P	STONE-bSSFP 3P	STONE-GRE 2P	STONE-GRE 3P
**Temperature**	151.8 (26.5)	78.9 (30.8)	91.1 (52.8)	108.2 (39.4)	98.3 (51.0)	116.4 (38.3)
**Session**	99.0 (73.5)	64.0 (32.2)	29.6 (28.5)	35.5 (19.2)	27.3 (26.0)	30.2 (20.4)
**Repetition**	N/A	52.5 (37.0)	0.0 (0.0)	0.0 (0.1)	0.00 (0.1)	0.0 (0.3)
**Slice**	N/A	N/A	9.0 (18.8)	22.1 (41.4)	9.9 (22.9)	26.9 (40.9)

In slice-interleaved T_1_ mapping, the main source of variability is temperature when reconstructed with a 2-parameter fit model, and slice when reconstructed with a 3-parameter fit. Variability due to repetition is minimal with variance decompositions less than 1%. SE, spin-echo; STONE-bSSFP, slice-interleaved T_1_ with balanced steady‐state free precession; STONE-GRE, slice-interleaved T_1_ with spoiled gradient echo.

#### Performance analysis against the spin echo

Between-session reproducibility and comparison of slice-interleaved T_1_ mapping sequences against SE T_1_ and MOLLI are summarized in Table 2. Slice-interleaved T_1_ mapping sequences provided superior between-session reproducibility compared to SE T_1_ when reconstructed with a 2-parameter fit model (*p*<0.05). There were no statistically significant differences between the slice-interleaved T_1_ and the reference when reconstructed with a 3-parameter fit model (*p*>0.05). Slice-interleaved T_1_ mapping sequences provided superior between-session reproducibility compared to MOLLI (*p*<0.05).

**Table 2:** Between-session reproducibility and the comparison of slice-interleaved T_1_ mapping sequences against SE T_1_ and MOLLI.

	SE T_1_	MOLLI	STONE-bSSFP 2P	STONE-bSSFP 3P	STONE-GRE 2P	STONE GRE 3P
**Between-session Reproducibility (CV, %)**	1.1±0.5	1.2±0.6	0.8±0.4	1.0±0.3	1.0±0.4	1.0±0.4
**p-value (vs. SE T**_**1**_**)**	N/A	N/A	0.005	0.377	0.001	0.117
**p-value (vs. MOLLI)**	N/A	N/A	0.011	0.031	0.010	0.024

Slice-interleaved T_1_ mapping sequences provided superior reproducibility compared to SE T_1_ when reconstructed with a 2-parameter fit model, and no statistically significant difference when reconstructed with a 3-parameter fit model. Slice-interleaved T_1_ mapping sequences provided superior reproducibility compared to MOLLI.

Slice-interleaved T_1_ mapping showed good agreement to the SE measurement with Pearson correlation coefficients of 1.00 (*p*<0.001) for all STONE-bSSFP 2P, STONE-GRE 2P, STONE-bSSFP 3P, and STONE-GRE. MOLLI also showed good agreement to the SE with Pearson correlation coefficients of 1.00 (*p*<0.001). All sequences showed good correlation to SE measurements with regression slopes as follows: MOLLI = 0.94 (95% CI: 0.936–0.945), STONE-bSSFP 2P = 0.95 (95% CI: 0.949–0.958), STONE-GRE 2P = 0.96 (95% CI: 0.957–0.961), STONE-bSSFP 3P = 0.97 (95% CI: 0.971–0.976), STONE-GRE 3P = 0.99 (95% CI: 0.987–0.992) (Table 3).

**Table 3:** Linear regression analysis of slice-interleaved T_1_ mapping sequences against reference SE T_1_ measurements.

	Regression Slope (Standard Error)	95% Confidence Interval
**MOLLI**	0.9407 (0.0023)	0.9362, 0.9452
**STONE-bSSFP 2P**	0.9536 (0.0024)	0.9489, 0.9583
**STONE-bSSFP 3P**	0.9737 (0.0011)	0.9715, 0.9759
**STONE-GRE 2P**	0.9591 (0.0011)	0.9569, 0.9613
**STONE GRE 3P**	0.9894 (0.0011)	0.9872, 0.9916

All sequences show strong agreement with the reference measurements with regression slopes of 0.9–1.0 and tight 95% confidence limits.

**Fig 4:**
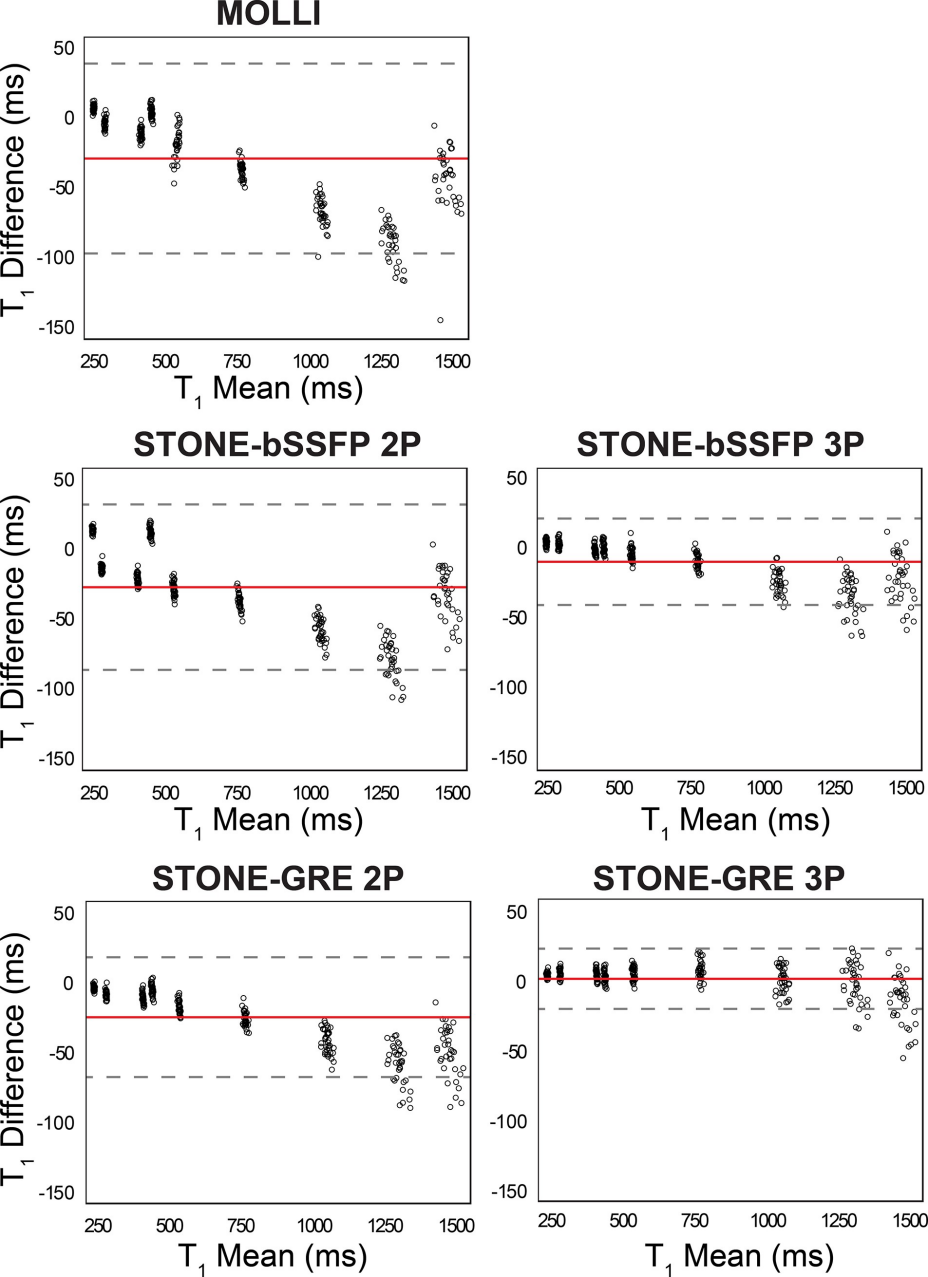
Bland-Altman analyses of slice-interleaved T_1_ mapping sequences against SE measurements. The mean difference (bias) is presented as the red line, and the 95% limits of agreement (mean ± 2 standard deviations) are presented as dashed lines. Each data point represents one study time point which was averaged for all repetitions and slices within each session. The T_1_ mapping sequences show underestimation compared to the reference measurement. STONE-GRE 3P shows strongest agreement with the reference measurement with an underestimation less than 1 ms.

Bland-Altman analysis results for all vials are shown in Fig 4, and the result per each vial is shown in S3 Table. STONE-GRE 3P showed very close T_1_ values to the SE with an underestimation less than 1 ms. T_1_ bias between SE and other T_1_ mapping sequences were as follows: MOLLI = -29.6 ms, STONE-bSSFP 2P = -27.9 ms, STONE-bSSFP 3P = -10.2 ms, STONE-GRE 2P = -25.7 ms. The % of data points outside the 95% limits of agreement were as follows: MOLLI = 3.7%, STONE-bSSFP 2P = 3.4%, STONE-bSSFP 3P = 5.6%, STONE-GRE 2P = 5.3%, STONE-GRE 3P = 5.3%.

### T_2_ mapping

#### Estimation of coefficient of variation

**Fig 5:**
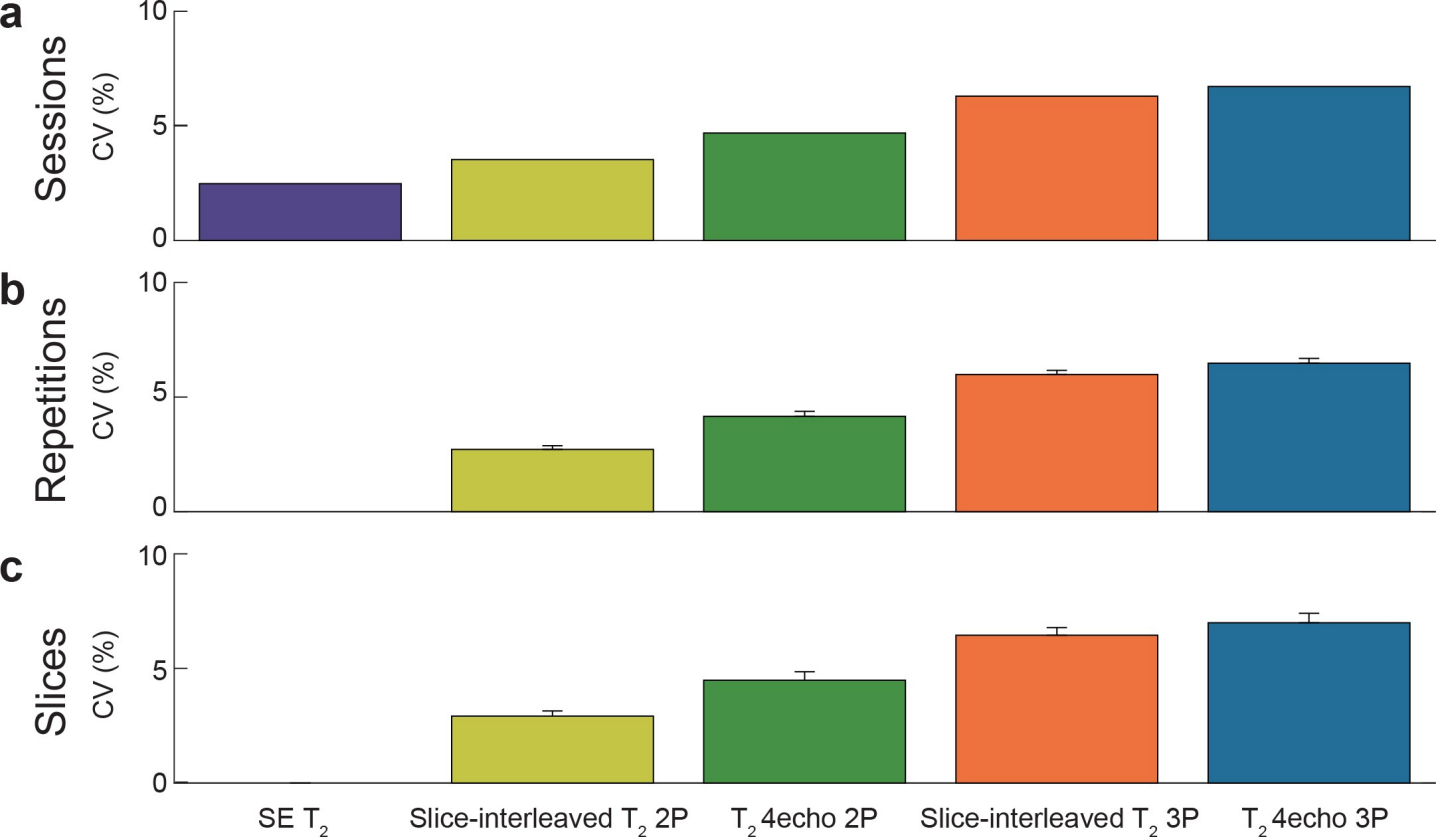
Reproducibility between sessions, and repeatability between repetitions and slices of slice-interleaved T_2_ mapping sequences were estimated using coefficients of variation (CV). Slice-interleaved T_2_ mapping had good between-session reproducibility (CV: SE T_2_ = 2.5%, slice-interleaved T_2_ 2P = 3.5%, slice-interleaved T_2_ 3P = 6.3%, slice-interleaved T_2_ 4-T_2_preps 2P = 4.7%, slice-interleaved T_2_ 4-T_2_preps 3P = 6.7%), between-repetition repeatability (CV: slice-interleaved T_2_ 2P = 2.7±0.2%, slice-interleaved T_2_ 3P was 6.0±0.2%, slice-interleaved T_2_ T_2_preps 2P = 4.2±0.2%, slice-interleaved T_2_ T_2_preps 3P was 6.5±0.2%), and good between-slice repeatability (CV: slice-interleaved T_2_ 2P = 2.9±0.2%, slice-interleaved T_2_ 3P = 6.5±0.3%, slice-interleaved T_2_ T_2_preps 2P = 4.5±0.4%, slice-interleaved T_2_ T_2_preps 3P was 7.0±0.4%).

High reproducibility between sessions, and high repeatability between repetitions and slices of slice-interleaved T_2_ mapping sequences were observed with a CV less than 7% (Fig 5).

#### Variance decomposition analysis

The sources of variabilities are summarized in Table 4. The main source of variability was the slice location/ orientation, which represents variability due to spatial location and B_0_, B_1_ field inhomogeneity. The second source of variability was the temperature. Variability in repeated measurements was minimal with variance decompositions of 0%.

**Table 4:** Sources of variability in T_2_ mapping defined by the variance decomposition analysis.

T_2_ Mapping, variance [ms^2^] (variance ratio [%])					
	SE T_2_	Slice-interleaved T_2_ 2P	Slice-interleaved T_2_ 3P	T_2_ 4echo 2P	T_2_ 4echo 3P
**Temperature**	1.8 (83.5)	2.2 (35.1)	1.4 (12.5)	2.0 (21.4)	1.3 (10.4)
**Session**	0.4 (16.5)	0.6 (9.7)	0.3 (3.0)	0.5 (5.0)	0.3 (2.4)
**Repetition**	N/A	0.0 (0.0)	0.0 (0.0)	0.0 (0.0)	0.0 (0.0)
**Slice**	N/A	3.4 (55.3)	9.3 (84.5)	7.0 (73.6)	10.9 (87.2)

In slice-interleaved T_2_ mapping, the main source of variability is slice, representing different spatial locations and different B_0_ and B_1_ field inhomogeneity. The variability in repeated measurements is minimal with variance decompositions of 0%.

#### Performance analysis against the spin echo

Slice-interleaved T_2_ mapping yielded lower between-session reproducibility than SE T_2_ (3.5 vs. 2.5% for slice-interleaved T_2_ 2P vs. SE T_2_; 6.3 vs. 2.5% for slice-interleaved T_2_ 3P vs. SE T_2_; 4.7 vs. 2.5% for T_2_ 4echo 2P vs. SE T_2_; 6.7 vs. 2.5% for T_2_ 4echo 3P vs. SE T_2_).

Slice-interleaved T_2_ mapping showed good correlation with Pearson correlation coefficients of 0.92 for slice-interleaved T_2_ 2P, 0.91 for slice-interleaved T_2_ 3P, 0.93 for T_2_ 4echo 2P, and 0.91 for T_2_ 4echo 3P (*p* <0.001 for all). Slice-interleaved T_2_ mapping showed good correlation to SE T_2_ with regression slopes as follows: slice-interleaved T_2_ 2P = 1.06 (95% CI: 0.91–1.21), slice-interleaved T_2_ 3P = 0.78 (95% CI: 0.66–0.90), T_2_ 4echo 2P = 1.04 (95% CI: 0.90–1.18), and T_2_ 4echo 3P = 0.78 (95% CI: 0.66–0.90) (Table 5).

**Table 5:** Linear regression of slice-interleaved T_2_ mapping sequences against the reference SE T_2_ measurements.

	Regression Slope (Standard Error)	95% Confidence Interval
**Slice-interleaved T**_**2**_ **2P**	1.0594 (0.0767)	0.9091, 1.2097
**Slice-interleaved T**_**2**_ **3P**	0.7830 (0.0606)	0.6642, 0.9018
**T**_**2**_ **4echo 2P**	1.0436 (0.0707)	0.9050, 1.1822
**T**_**2**_ **4echo 3P**	0.7828 (0.0623)	0.6607, 0.9049

Slice-interleaved T_2_ sequences show good agreement with reference measurements with regression slopes of 0.8–1.1.

**Fig 6:**
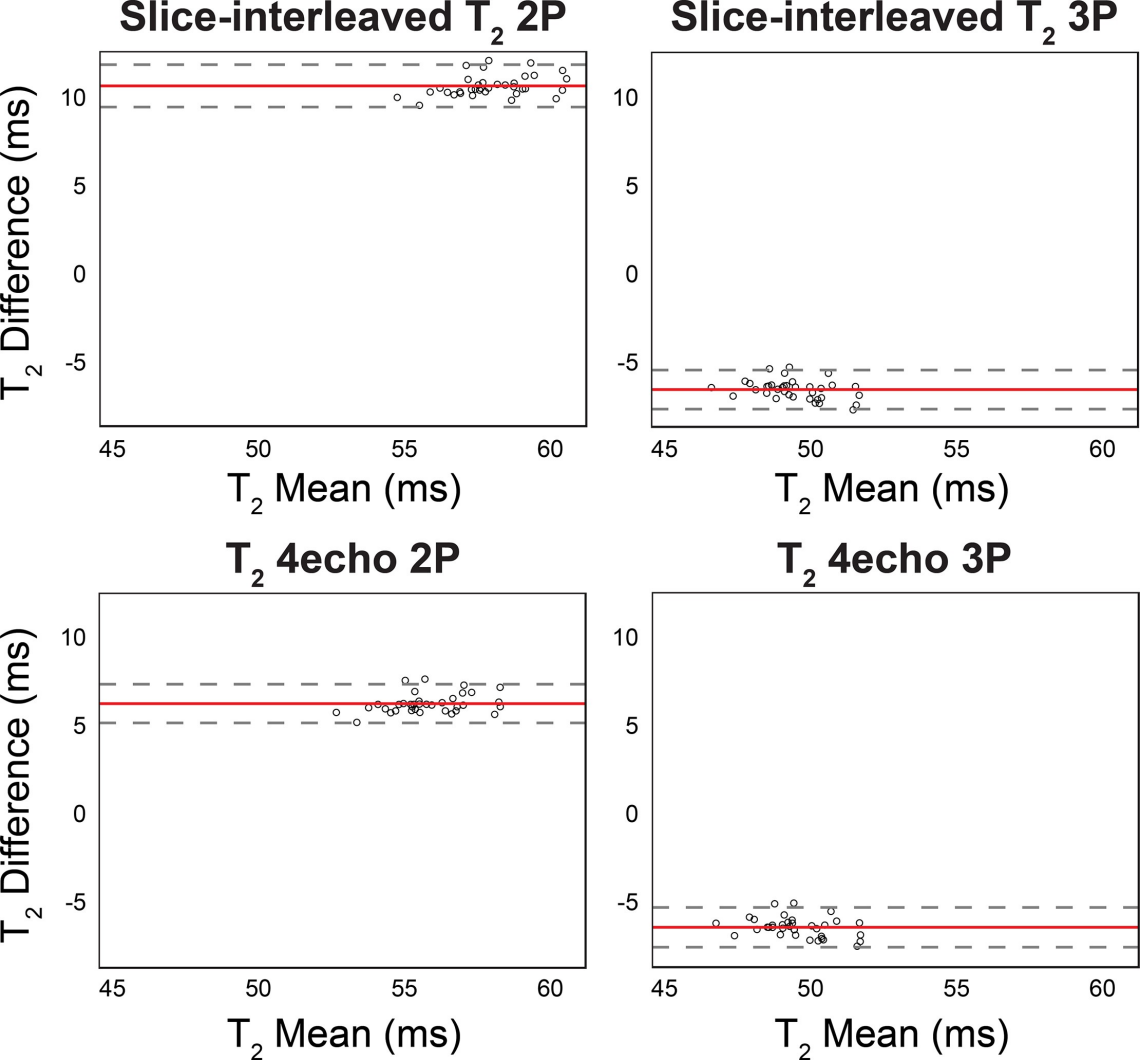
Bland-Altman plots of slice-interleaved T_2_ mapping sequences against the reference SE T_2_ measurements. Bland-Altman analyses of slice-interleaved T_2_ mapping shows an overestimation when the map is reconstructed with a 2-parameter fit model, and an underestimation when reconstructed with a 3-parameter fit model. Each data point represents one study time point which was averaged for all repetitions and slices within session. The mean difference (bias) is presented as the red line, and the 95% limits of agreement (mean ± 2 standard deviations) are presented as dashed lines.

Bland-Altman analysis of slice-interleaved T_2_ mapping sequences showed different estimation biases depending on the fitting model (Fig 6). The slice-interleaved T_2_ showed overestimation when reconstructed with a 2-parameter fit model (slice-interleaved T_2_ 2P = 10.6 ms, T_2_ 4echo 2P = 6.3 ms), and an underestimation when reconstructed with a 3-parameter fit model (slice-interleaved T_2_ 3P = -6.4 ms, T_2_ 4echo 3P = -6.3 ms) against the SE T_2_ (Fig 6). The % of data points outside the 95% limits of agreement were as follows: slice-interleaved T_2_ 2P = 5.6%, slice-interleaved T_2_ 3P = 8.3%, T_2_ 4echo 2P = 5.6%, T_2_ 4echo 3P = 5.6%.

## Discussion

In this study, we demonstrate highly reproducible long-term measurements of slice-interleaved T_1_ and T_2_ mapping with a CV less than 2% for T_1_ and less than 7% for T_2_. Reproducible measurements are essential to detect subtle changes in T_1_ and T_2_ times due to pathological processes. In particular, assessing long-term measurement stability is necessary for confidently differentiating variability due to disease progression or treatment efficacy over an extended period in a longitudinal study. The current phantom study reports rigorous long-term technical performance of slice-interleaved T_1_ and T_2_ mapping sequences to better understand baseline variations under controlled conditions.

Regular phantom-based quality control is recommended to ensure stability of a CMR system. Our study reports baseline long term variability which can be used to assess the stability of a CMR system for quality control, and to establish normal and clinical values with expected ranges of variability due to technical confounders. In the long-term time span, factors such as scanner performance can result in systematic differences compared to a shorter time interval. Furthermore, phantom-based quality control allows for T_1_ or T_2_ accuracy assessment and temperature sensitivity measurements as monitored for each session in our study.

T_1_ and T_2_ measurements with identical imaging parameters can still vary across session, repetition, and slice due to various factors. Our study showed that the main source of variability in T_1_ mapping was temperature when reconstructed with a 2-parameter fit model, and slice when reconstructed with a 3-parameter fit model. Temperature impacts diffusion coefficients [44], which can in turn impact T_1_ and T_2_. In vials with longer T_1_ times where a concentration of Ni^2+^ is low, T_1_ becomes more sensitive to temperature due to the temperature sensitivity of the T_1_ of water in gel [38]. Imperfect inversion pulses due to field inhomogeneity can be modeled using a 3-parameter fit model [9, 30, 45]. In turn, variability due to slice, representative of B_0_, B_1_ inhomogeneity, becomes dominant. For T_2_ mapping, slice was the main source of variability, which may be associated with differences in B_0_ and B_1_ field inhomogeneity. Variability in repeated measurements was negligible.

In this phantom study, we used in-vivo protocols currently used in our laboratory to mimic a clinically-relevant setting. Identical in-plane resolution was used to maintain similar TEs for similar performance. We used extra padding around the phantom to create distance from the RF coils to approximate coil geometry and proximity when imaging the human heart. Slice-interleaved T_2_ mapping was acquired with 10 T_2_prep echo times, previously evaluated in-vivo [20, 22, 32].

The variability observed in the current study shows a similar CV magnitude range as that shown in in-vivo reproducibility studies. Recent shorter-term reproducibility studies in T_1_ mapping yield CV magnitude ranges similar to our results, where the CV of ShMOLLI and MOLLI are reported as 2% for 35 patients undergoing repeated measurements the following day [46]. Slice-interleaved T_1_ and T_2_ show between-day CVs of 2.1% and 6.3%, respectively, in 11 healthy subjects on a 2-day test/retest study [22]. Higher variation is expected in the in-vivo study performed in a longer time span over multiple sessions due to patient-related artifacts such as respiratory and cardiac motion.

We performed long-term between-session reproducibility assessment including SE measurements. Even though SE is typically used as the reference, no study has evaluated its measurement variability over an extended period. Between-session reproducibility of SE measurements was excellent, and slice-interleaved T_1_ mapping sequences showed superior between-session reproducibility compared to SE. In particular, STONE-GRE 3P had excellent agreement to SE with similar reproducibility and an underestimation of only < 1ms. Considering the long scan time of SE sequences (typically 5–6 hours for T_1_ and 1–2 hours for T_2_), alternative sequences for the reference measurement are desirable.

We performed Bland-Altman analyses in each individual vial and for all vials to reflect the unique dependence of T_1_ on the bias. Longer T_1_ times corresponded with higher T_1_ error as previously reported [45]. We studied the measurement variability of slice-interleaved T_1_ and T_2_ maps reconstructed using both 2-parameter and 3-parameter fit models. For all sequences, higher reproducibility and repeatability was achieved when reconstructed with the 2-parameter fit model; however, the measurement bias was smaller when reconstructed with the 3-parameter fit model. This is in line with previous studies showing higher accuracy but lower precision when fitted with additional parameters [45]. Previous study demonstrated higher precision and reproducibility is achieved by increasing the number of T_2_prep echo times from 3 to 14, where the effect nearly saturates above 10 echo times in both phantom and in-vivo studies [47]. Our result shows higher reproducibility for T_2_ mapping with 9 T_2_prep images compared to 4 T_2_prep images as previously reported. We observed higher variability in T_2_ mapping, which may be due to lower SNR of the T_2_prep sequence due to field inhomogeneities and spoiling gradient.

Our study has several limitations. We studied slice-interleaved T_1_ and T_2_ mapping sequences on a single MRI scanner at a field strength of 1.5 T. The T1MES phantom used in this study is not optimally designed for studying T_2_ mapping; therefore, T_2_ analysis was carried out in a single vial with similar myocardial T_1_ and T_2_ values. A phantom with a different T_2_ range needs to be developed to study T_2_ reproducibility. The CPMG SE used as a reference of T_2_ measurements may be susceptible to stimulated-echo related bias. Our data shows 10.6±1.5% T_2_ difference compared to the T_2_ measurements by slow SE acquired with 8 TEs from 10–640 ms [38]. We did not study the impact of SNR, although with a relatively large region of interest in the current study, the impact may be negligible. Respiratory and cardiac motion could degrade T_1_ and T_2_ mapping reproducibility and were not simulated in our phantom study. Future long-term reproducibility studies in humans are warranted to enhance our understanding of measurement variability in a more clinically relevant setting.

## Conclusions

Slice-interleaved T_1_ and T_2_ mapping sequences demonstrate highly reproducible measurement with a coefficient of variation less than 2% for T_1_, and 7% for T_2_ ranges of < 100 ms measured beyond one year. Slice-interleaved T_1_ mapping offers superior reproducibility than both MOLLI and SE T_1_ when reconstructed with a 2-paremeter fit model, and slice-interleaved T_2_ mapping shows lower reproducibility than SE T_2_. All sequences demonstrate strong agreement with reference SE measurements.

## Supporting information

S1 Fig Graphical illustration of the selected ROI on top of a weighted image. (DOCX)

S2 Fig Representative examples of T_1_ and T_2_ maps and weighted images for all sequences. (DOCX)

S3 Fig T_1_ measurements over 20 months in all 9 vials. (DOCX)

S4 Fig T_2_ measurements over 20 months in vial ‘F’. (DOCX)

S1 Table T_1_ measurements over 20 months in all vials. (DOCX)

S2 Table T_2_ measurements over 20 months in vial ‘F’. (DOCX)

S3 Table Bland-Altman analyses performed per each vial for T_1_ mapping. (DOCX)
