# Supplementary material for: Measurement reproducibility of slice-interleaved T1 and T2 mapping sequences over 20 months: A single center study
Source: PLoS One. 2019 Jul 25;14(7):e0220190. doi: 10.1371/journal.pone.0220190 (PMC6658153; doi:10.1371/journal.pone.0220190)
Supplement: S1 Fig — (DOCX) [file pone.0220190.s001.docx]

*

*

**S1 Fig.** Graphical illustration of the selected ROI on top of a weighted image. ROI was drawn once for each vial and was programmatically applied to the rest of the sequences and experiments. SE ROI was separately drawn once for SE T_1_ and programmatically applied to SE T_2_ and the rest of the experiments. ROI was contoured to include the central 50% of the area within each vial.
