# Supplementary material for: Measurement reproducibility of slice-interleaved T1 and T2 mapping sequences over 20 months: A single center study
Source: PLoS One. 2019 Jul 25;14(7):e0220190. doi: 10.1371/journal.pone.0220190 (PMC6658153; doi:10.1371/journal.pone.0220190)
Supplement: S2 Fig — (DOCX) [file pone.0220190.s002.docx]

**S2 Fig.** Representative examples of T_1_ and T_2_ maps and weighted images for all sequences.
