# Supplementary material for: Measurement reproducibility of slice-interleaved T1 and T2 mapping sequences over 20 months: A single center study
Source: PLoS One. 2019 Jul 25;14(7):e0220190. doi: 10.1371/journal.pone.0220190 (PMC6658153; doi:10.1371/journal.pone.0220190)
Supplement: S3 Fig — (DOCX) [file pone.0220190.s003.docx]

*
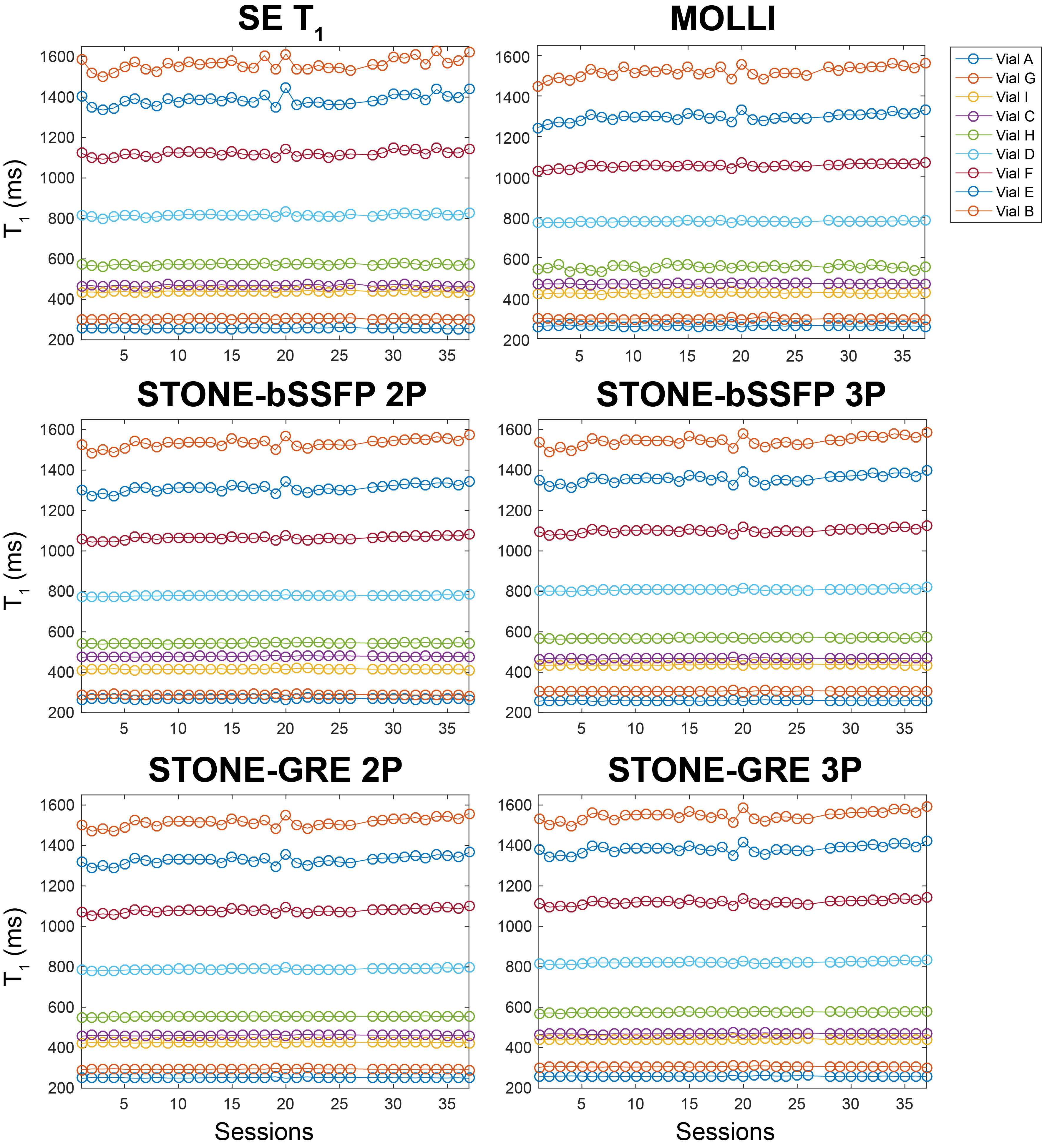
*

**S3 Fig.** T_1_ measurements over 20 months in all 9 vials. No systematic drift in the T_1_ measurements was found in 20 months. For STONE sequences, T_1_ was averaged over slices and repetitions for each session. Session 27 was excluded from analysis due to the incomplete acquisition of the SE T_1_ sequence.
