# Supplementary material for: Measurement reproducibility of slice-interleaved T1 and T2 mapping sequences over 20 months: A single center study
Source: PLoS One. 2019 Jul 25;14(7):e0220190. doi: 10.1371/journal.pone.0220190 (PMC6658153; doi:10.1371/journal.pone.0220190)
Supplement: S4 Fig — (DOCX) [file pone.0220190.s004.docx]

*
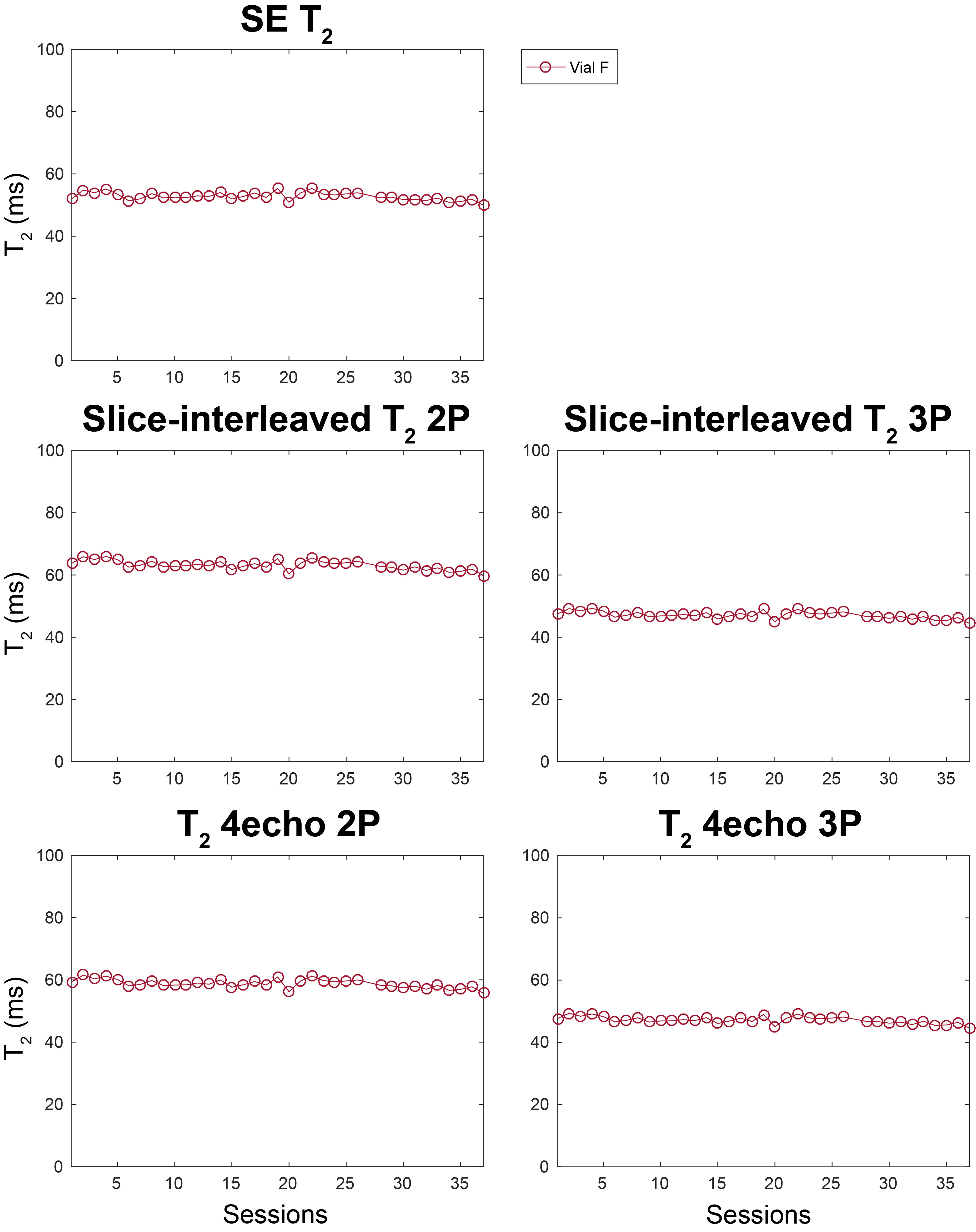
*

**S4 Fig.** T_2_ measurements over 20 months in vial ‘F’. No systematic drift in the T_2_ measurements was found in 20 months. For slice-interleaved T_2_ sequences, T_2_ was averaged over slices and repetitions for each session. Session 27 was excluded from the analysis due to the incomplete acquisition of the SE T_1_ sequence.
