# Supplementary material for: Measurement reproducibility of slice-interleaved T1 and T2 mapping sequences over 20 months: A single center study
Source: PLoS One. 2019 Jul 25;14(7):e0220190. doi: 10.1371/journal.pone.0220190 (PMC6658153; doi:10.1371/journal.pone.0220190)
Supplement: S1 Table — (DOCX) [file pone.0220190.s005.docx]

|  | **SE T_1_** | | | **MOLLI** | | |
| --- | --- | --- | --- | --- | --- | --- |
| **Vial** | **Slope** | **95% CI** | **p-vale** | **Slope** | **95% CI** | **p-vale** |
| **A** | 0.01 | -0.05 — 0.08 | 0.69 | 0 | -0.06 — 0.05 | 0.91 |
| **G** | 0.03 | -0.05 — 0.1 | 0.48 | 0 | -0.08 — 0.08 | 0.97 |
| **I** | 0.08 | -0.02 — 0.18 | 0.14 | 0.09 | 0.01 — 0.17 | 0.03 |
| **C** | 0.07 | -0.06 — 0.19 | 0.32 | 0.09 | 0.03 — 0.15 | < 0.001 |
| **H** | 0.17 | 0.04 — 0.31 | 0.02 | 0.24 | -0.02 — 0.49 | 0.08 |
| **D** | 0.33 | 0.14 — 0.52 | < 0.001 | 0.16 | 0.08 — 0.24 | < 0.001 |
| **F** | 0.7 | 0.31 — 1.10 | < 0.001 | 0.7 | 0.52 — 0.89 | < 0.001 |
| **E** | 1.22 | 0.50 — 1.95 | < 0.001 | 1.16 | 0.78 — 1.55 | < 0.001 |
| **B** | 1.39 | 0.68 — 2.10 | < 0.001 | 1.57 | 1.06 — 2.09 | < 0.001 |
|  | **STONE-bSSFP 2P** | | | **STONE-bSSFP 3P** | | |
| **A** | 0 | -0.02 — 0.02 | 0.87 | -0.02 | -0.04 — 0 | 0.06 |
| **G** | 0.01 | -0.01 — 0.03 | 0.33 | 0.05 | 0.03 — 0.08 | < 0.001 |
| **I** | 0.03 | 0.01 — 0.05 | 0.01 | 0.08 | 0.05 — 0.11 | < 0.001 |
| **C** | 0.08 | 0.07 — 0.10 | < 0.001 | 0.07 | 0.04 — 0.09 | < 0.001 |
| **H** | 0.11 | 0.08 — 0.14 | < 0.001 | 0.17 | 0.13 — 0.22 | < 0.001 |
| **D** | 0.18 | 0.16 — 0.20 | < 0.001 | 0.28 | 0.25 — 0.32 | < 0.001 |
| **F** | 0.59 | 0.53 — 0.65 | < 0.001 | 0.66 | 0.58 — 0.74 | < 0.001 |
| **E** | 1.11 | 0.99 — 1.22 | < 0.001 | 1.21 | 1.07 — 1.35 | < 0.001 |
| **B** | 1.23 | 1.11 — 1.35 | < 0.001 | 1.25 | 1.12 — 1.38 | < 0.001 |
|  | **STONE-GRE 2P** | | | **STONE-GRE 3P** | | |
| **A** | 0.02 | 0.00 — 0.03 | 0.04 | 0.02 | 0.00 — 0.04 | 0.02 |
| **G** | 0 | -0.01 — 0.02 | 0.74 | 0.02 | 0 — 0.05 | 0.07 |
| **I** | 0.04 | 0.03 — 0.05 | < 0.001 | 0.05 | 0.03 — 0.07 | < 0.001 |
| **C** | 0.08 | 0.06 — 0.10 | < 0.001 | 0.09 | 0.07 — 0.11 | < 0.001 |
| **H** | 0.12 | 0.10 — 0.15 | < 0.001 | 0.15 | 0.12 — 0.18 | < 0.001 |
| **D** | 0.29 | 0.26 — 0.32 | < 0.001 | 0.37 | 0.33 — 0.42 | < 0.001 |
| **F** | 0.64 | 0.57 — 0.70 | < 0.001 | 0.67 | 0.60 — 0.75 | < 0.001 |
| **E** | 1.05 | 0.93 — 1.17 | < 0.001 | 1.08 | 0.93 — 1.22 | < 0.001 |
| **B** | 1.25 | 1.13 — 1.37 | < 0.001 | 1.29 | 1.16 — 1.43 | < 0.001 |

**S1 Table**. T_1_ measurements over 20 months in all vials. No systematic drift in the vials with short T_1_ (<1000 ms) with regression slope near 0. Increased T_1_ measurement was observed in vials with long T_1_ (>1000 ms) with regression slope near 1.
