# Supplementary material for: Measurement reproducibility of slice-interleaved T1 and T2 mapping sequences over 20 months: A single center study
Source: PLoS One. 2019 Jul 25;14(7):e0220190. doi: 10.1371/journal.pone.0220190 (PMC6658153; doi:10.1371/journal.pone.0220190)
Supplement: S2 Table — (DOCX) [file pone.0220190.s006.docx]

|  | **SE T_2_** | | |  | | |
| --- | --- | --- | --- | --- | --- | --- |
| **Vial** | **Slope** | **95% CI** | **p-vale** | **Slope** | **95% CI** | **p-vale** |
| **F** | -0.05 | -0.09 — -0.01 | 0.01 |  |  |  |
|  | **Multi-slice T_2_ 2P** | | | **Multi-slice T_2_ 3P** | | |
| **F** | -0.08 | -0.1 — -0.07 | < 0.001 | -0.06 | -0.08 — -0.04 | < 0.001 |
|  | **T_2_ 4echo 2P** | | | **T_2_ 4echo 3P** | | |
| **F** | -0.07 | -0.1 — -0.05 | < 0.001 | -0.06 | -0.08 — -0.03 | < 0.001 |

**S2 Table**. T_2_ measurements over 20 months in vial ‘F’. No systematic drift in the T_2_ measurements was found over the 20 month study duration with regression slope near 0.
