# Supplementary material for: Measurement reproducibility of slice-interleaved T1 and T2 mapping sequences over 20 months: A single center study
Source: PLoS One. 2019 Jul 25;14(7):e0220190. doi: 10.1371/journal.pone.0220190 (PMC6658153; doi:10.1371/journal.pone.0220190)
Supplement: S3 Table — (DOCX) [file pone.0220190.s007.docx]

|  | **MOLLI** | **STONE-bSSFP 2P** | **STONE-bSSFP 3P** | **STONE-GRE 2P** | **STONE GRE 3P** |
| --- | --- | --- | --- | --- | --- |
| **Vial ‘A’** | | | | | |
| **T_1_ Difference** | 5.6 | 11.6 | 2.8 | -5.4 | 1.6 |
| **95% Lower Limit** | 0.9 | 7.8 | -1.9 | -8.9 | -2.3 |
| **95% Upper Limit** | 10.3 | 15.4 | 7.5 | -1.9 | 5.5 |
| **Vial ‘G’** | | | | | |
| **T_1_ Difference** | -5.1 | -14.9 | 2.1 | -9.7 | 3.0 |
| **95% Lower Limit** | -12.7 | -20.1 | -4.0 | -15.0 | -3.3 |
| **95% Upper Limit** | 2.5 | -9.6 | 8.1 | -4.4 | 9.3 |
| **Vial ‘I’** | | | | | |
| **T_1_ Difference** | -12.3 | -22.9 | -2.3 | -12.6 | 2.3 |
| **95% Lower Limit** | -19.9 | -30.4 | -8.5 | -19.8 | -4.9 |
| **95% Upper Limit** | -4.6 | -15.3 | 3.9 | -5.3 | 9.5 |
| **Vial ‘C’** | | | | | |
| **T_1_ Difference** | 2.9 | 10.4 | -0.1 | -6.6 | 0.6 |
| **95% Lower Limit** | -5.3 | 2.8 | -8.0 | -14.6 | -7.6 |
| **95% Upper Limit** | 11.2 | 18.1 | 7.9 | 1.5 | 8.9 |
| **Vial ‘H’** | | | | | |
| **T_1_ Difference** | -19.1 | -28.9 | -4.6 | -18.5 | 2.8 |
| **95% Lower Limit** | -40.4 | -38.8 | -14.4 | -27.6 | -6.8 |
| **95% Upper Limit** | 2.2 | -19.1 | 5.3 | -9.4 | 12.3 |
| **Vial ‘D’** | | | | | |
| **T_1_ Difference** | -37.4 | -37.7 | -9.7 | -27.1 | 5.2 |
| **95% Lower Limit** | -49.0 | -48.9 | -20.1 | -37.3 | -7.8 |
| **95% Upper Limit** | -25.8 | -26.4 | 0.8 | -16.9 | 18.3 |
| **Vial ‘F’** | | | | | |
| **T_1_ Difference** | -66.1 | -57.6 | -24.4 | -42.9 | -1.5 |
| **95% Lower Limit** | -86.6 | -74.7 | -40.5 | -59.9 | -19.3 |
| **95% Upper Limit** | -45.7 | -40.4 | -8.2 | -25.8 | 16.3 |
| **Vial ‘E’** | | | | | |
| **T_1_ Difference** | -90.6 | -78.1 | -33.9 | -58.8 | -3.8 |
| **95% Lower Limit** | -126.3 | -105.0 | -61.0 | -87.0 | -32.3 |
| **95% Upper Limit** | -54.9 | -51.2 | -6.8 | -30.6 | 24.7 |
| **Vial ‘B’** | | | | | |
| **T_1_ Difference** | -44.0 | -33.3 | -22.0 | -50.1 | -16.0 |
| **95% Lower Limit** | -89.5 | -67.6 | -55.5 | -84.7 | -49.9 |
| **95% Upper Limit** | 1.6 | 1.1 | 11.6 | -15.4 | 17.9 |

**S3 Table**. Bland-Altman analyses performed per each vial for T_1_ mapping. The mean T_1_ difference (bias) and the 95% limits of agreement (mean ± 2 standard deviations) are presented for each sequence for each vial.
